# TBC1D3 family is a prognostic biomarker and correlates with immune infiltration in kidney renal clear cell carcinoma

**DOI:** 10.1016/j.omto.2021.06.014

**Published:** 2021-07-10

**Authors:** Bei Wang, Dandan Chen, Haiying Hua

**Affiliations:** 1 Department of Institute of Integration of Traditional Chinese and Western Medicine, the Affiliated Hospital of Jiangnan University, Wuxi 124122, China; 2 Department of Education, School of Humanities, Jiangnan University, Wuxi 124122, China; 3 Department of Hematology, the Affiliated Hospital of Jiangnan University, Wuxi 124122, China

**Keywords:** TBC1D3, kidney clear cell carcinoma, infiltrating immune cell, prognosis marker, tumor-infiltrating lymphocytes

## Abstract

The TBC1D3 family is overexpressed in many cancers, including kidney renal clear cell carcinoma (KIRC), which is associated with tumor-infiltrating lymphocytes. However, the expression and prognosis of TBC1D3 family and tumor-infiltrating lymphocytes in KIRC remain unknown. In the present study, we systematically explored and validated the expression and prognostic value of TBC1D3 family expression in KIRC using multiple public databases. In addition, the function of the TBC1D3 family members and the correlations between TBC1D3 family expression and KIRC immune infiltration levels were investigated. We found that TBC1D3 family members were rarely mutated (less than 5 frequencies). TBC1D3 family was overexpressed in KIRC; high expression of the TBC1D3 family members was correlated with poor prognosis. In addition, TBC1D3D may positively regulate proliferation, and overexpression of TBC1D3 promoted clear cell renal cell carcinoma proliferation *in vitro*. In terms of immune infiltrating levels, TBC1D3 family expression was positively associated with CD4^+^ T cells infiltrating levels. These findings suggest that the TBC1D3 family expression is correlated with prognosis and immune infiltrating levels. Therefore, the TBC1D3 family can be used as a biomarker for KIRC and a prognostic biomarker for determining the prognosis and immune infiltration levels in KIRC.

## Graphical abstract

**Figure:**
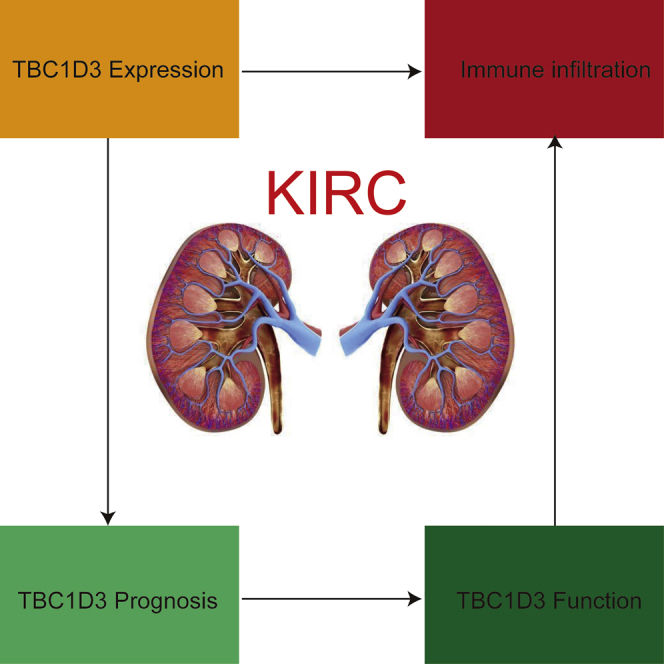

## Teaser

The TBC1D3 family can be used as a biomarker for kidney renal clear cell carcinoma (KIRC) and a prognostic biomarker. In addition, TBC1D3 promotes the cell proliferation and correlates with immune infiltration levels and T cell exhaustion in KIRC.

## Introduction

Clear cell renal cell carcinoma (ccRCC), or kidney renal clear cell carcinoma (KIRC), is the most common malignant tumor of renal cancer, accounting for 75%–82% of primary malignancies in the kidney.^1^ In a variety of clinical and genomic studies, KIRC has been shown to be a highly immune-infiltrated tumor, and KIRC is one of the earliest malignancies to respond to immune therapy.^2^

TBC1D3 is a member of the TBC1 domain family and a hominoid-specific gene, which is amplified on chromosome 17.^3^^,^^4^ TBC1D3 represents a family of molecules encoded by eight paralogs (A–H).^5^ TBC1D3 is expressed in human tissues and overexpressed in prostate, breast, pancreatic, and bladder cancers, as well as in myelodysplatic syndrome. TBC1D3 acts as a GTPase-activating protein for RAB5, and TBC1D3 is identified as a novel nucleocytoplasmic protein, which is regulated by the microtubule network.^6^ Recently, TBC1D3 was found to promote breast cancer cell migration.^7^ However, the function of the TBC1D3 family in KIRC remains unknown. Therefore, we explore the expression, prognosis, and tumor infiltrating lymphocytes of the TBC1D3 family in KIRC through multiple databases.

In this present study, we analyzed the expression and prognostic value of the TBC1D3 family in KIRC using public online databases: Gene Set Cancer Analysis (GSCA), UALCAN, and Kaplan-Meier plotter. TBC1D3 family function in KIRC was performed by CancerSEA. We investigated the association of TBC1D3 family expression and immune inhibitor, as well as its association with infiltrating immune cell using the TISIDB and TIMER. This is the first comprehensive study of associations between the expression of TBC1D3 family genes and their clinical, molecular, and immunological characteristics in KIRC. Our results may help to optimize immunotherapy for patients with kidney clear cell carcinoma.

## Results

### Expression of the TBC1D3 family in kidney renal clear cell carcinoma

**Figure 1:**
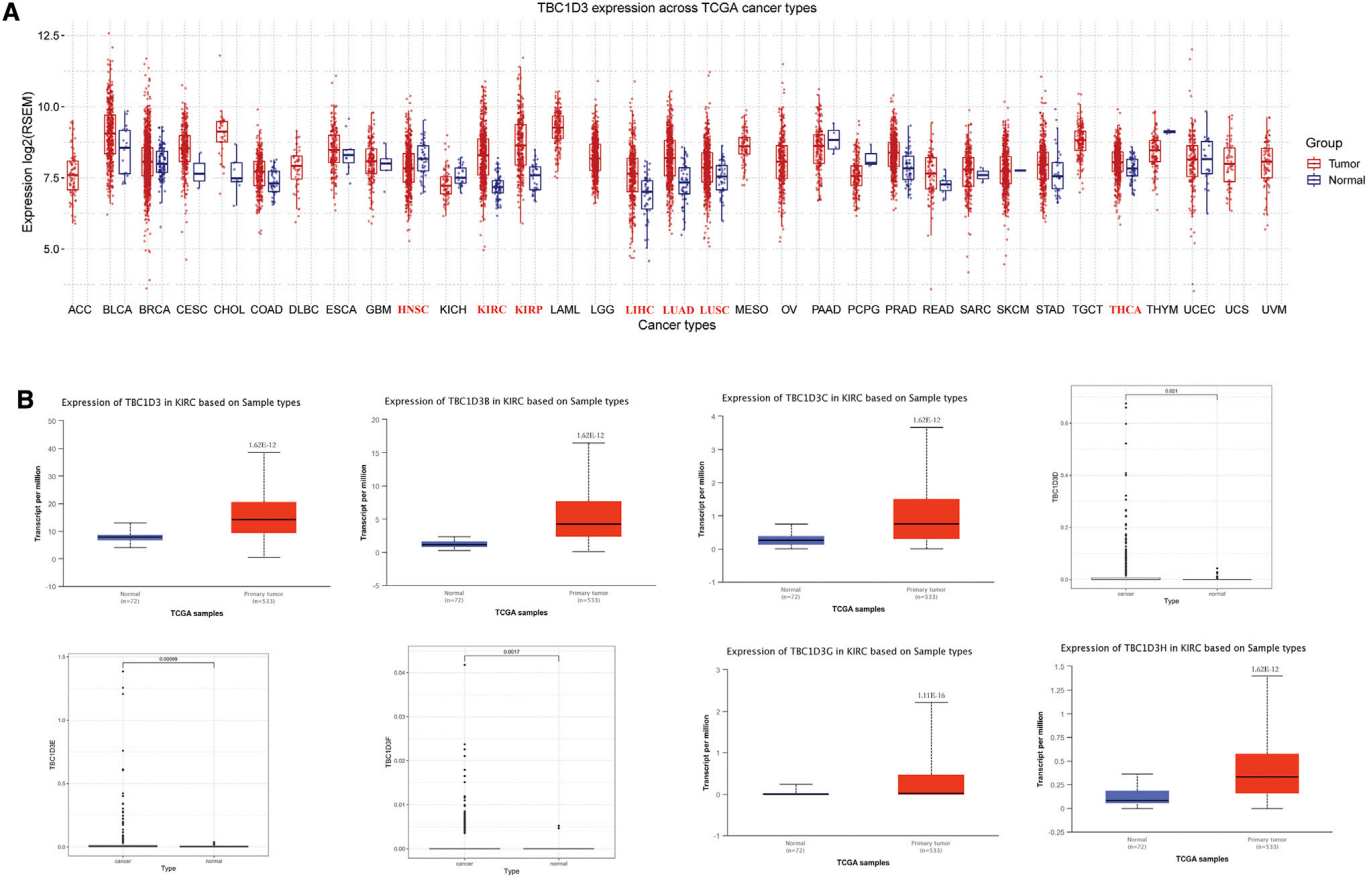
Expression of TBC1D3 family in KIRC and normal tissue (A) TBC1D3 expression levels in different tumor types from TCGA database were determined by GSCA. Significant differences were shown in red. (B) TBC1D3 family expression in KIRC is significantly higher than that in normal tissue from UALCAN and R script.

To investigate the differences of TBC1D3 expression between tumors and normal tissues in various types of cancers, the TBC1D3 mRNA levels were analyzed using GSCA. The results showed that the TBC1D3 expression was respectively higher in KIRC, KIRP (kidney renal papillary cell carcinoma), LIHC (liver hepatocellular), LUAD (lung adenocarcinoma), LUSC (lung squamous cell carcinoma), and THCA (thyroid carcinoma). In addition, lower expression was observed in HNSC (head and neck squamous cell carcinoma) (Figure 1A). The differential expression of TBC1D3 family members in KIRC cohort tumor and non-tumor tissues was analyzed by using UALCAN. Information of TBC1D3D/TBC1D3E/TBC1D3F was not found in UALCAN. We download the original file of TCGA and analyzed TBC1D3D/TBC1D3E/TBC1D3F expression. The expression levels of the TBC1D3 family were remarkably higher in KIRC than in normal tissues (Figure 1B). Furthermore, the expression of the TBC1D3 family was associated with a patient’s race, gender, age, tumor grade, and nodal metastasis status (Table S1). To verify the above conclusions, we performed real-time PCR in 10 pairs of matched KIRC tissues and adjacent normal tissues. The TBC1D3 mRNA expression was highly expressed in KIRC tissues, compared with adjacent normal tissues (Figure S1).

### Genomic alterations of the TBC1D3 family and gene and protein network

**Figure 2:**
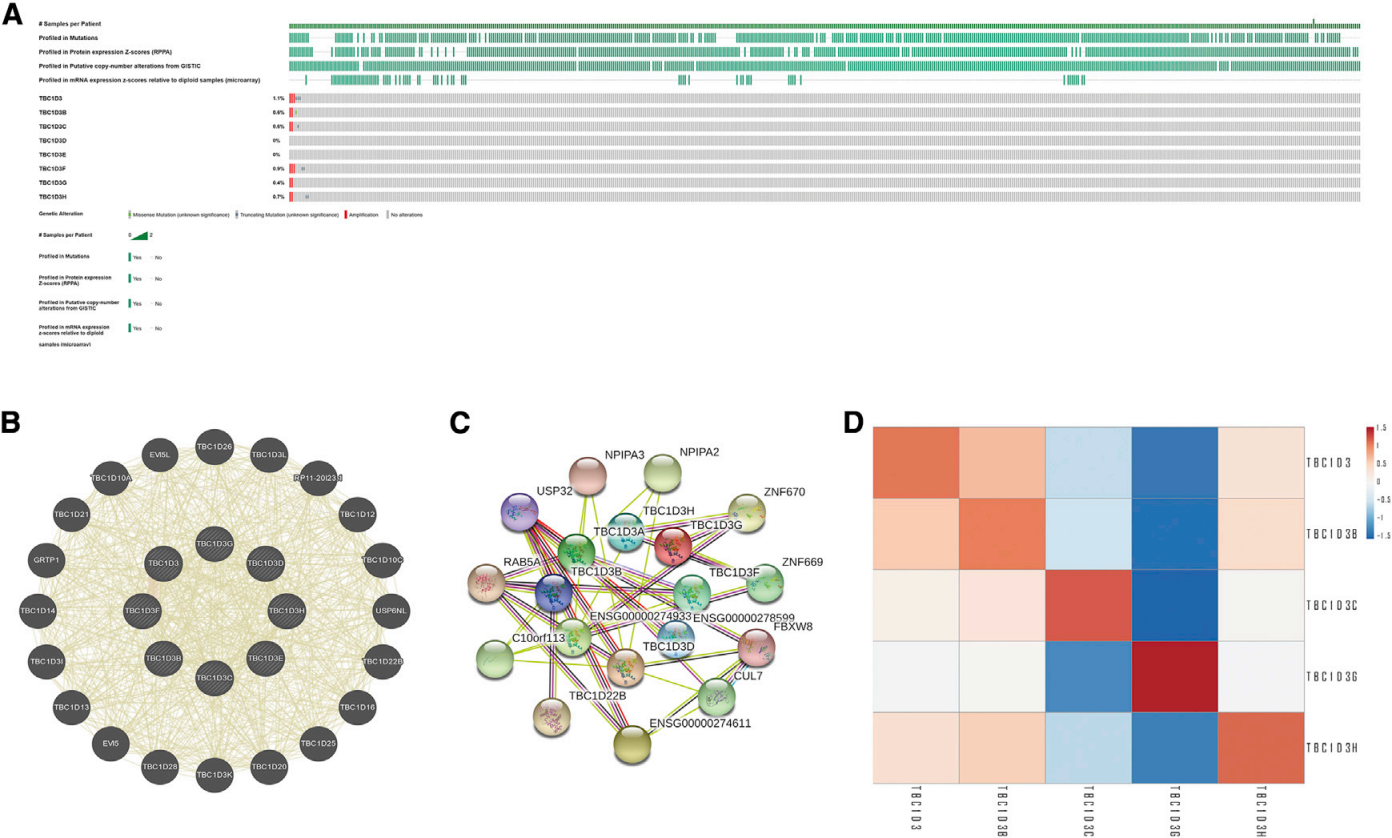
Genomic alterations of TBC1D3 family members and gene-gene and protein-protein interaction network of genes of the TBC1D3 family (A) The OncoPrint schematic provides an overview of genomic alterations of the TBC1D3 family in KIRC from TCGA. (B) The gene network associated with the TBC1D3 gene family, drawn by using GeneMANIA. (C) A network diagram of interactions between proteins encoded by genes of the TBC1D3 family, drawn by using STRING. (D) The correlation analysis for the TBC1D3 family members.

We then used the cBioportal to determine the types and frequencies of TBC1D3 family alterations in the TCGA KIRC samples. Results showed that TBC1D3 family members were rarely mutated (less than 5 frequencies), which was highly conserved (Figure 2A). Then, we used “correlation analysis” by cBioportal for five members of the TBC1D3 family, which showed that associations among TBC1D3 family members were positively correlated, except TBC1D3C, which was negatively correlated with TBC1D3G (Figure 2D). The gene-gene and protein-protein interaction network, which was generated by using GeneMANIA and STRING showed that 20 potential target genes and 11 potential target proteins interacted with the TBC1D3 family (Figures 2B and 2C).

### Prognostic potential of the TBC1D3 family in KIRC

**Figure 3:**
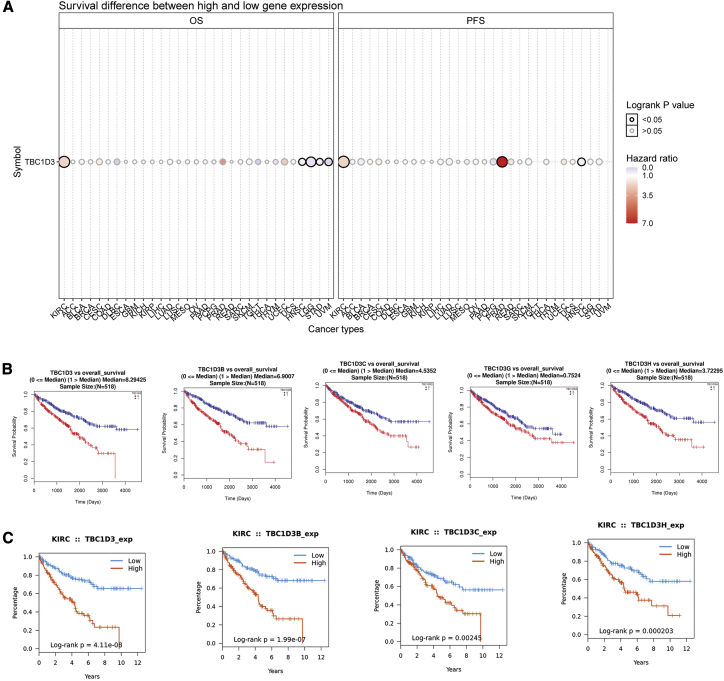
The prognostic value of TBC1D3 family members in KIRC (A) Survival difference between high and low expression of TBC1D3 across 33 cancer types from TCGA. (B) Survival curves of TBC1D3 family members were analyzed by LinkedOmics. (C) Survival curves of TBC1D3 family were analyzed by TISIDB.

We next investigated whether TBC1D3 family expression was associated with KIRC prognosis. The impact of TBC1D3 family expression on survival rates was evaluated using GSCA, TISIDB, LinkedOmics, and Kaplan-Meier plotter. GSCA analysis revealed that TBC1D3 expression was positively correlated with overall survival (OS) and progress free survival in KIRC among multiple tumors. Besides, higher expression of TBC1D3 had a short survival (Figure 3A). OS analysis from TISIDB and LinkedOmics indicated that patients with high expression of the TBC1D3 family members had a short OS (Figures 3B and 3C). To further explore the contribution of TBC1D3 to the clinical characteristics of KIRC, we investigated the association between TBC1D3 expression and clinicopathological characteristics using the Kaplan-Meier plotter. As shown in Table 1, high TBC1D3 and TBC1D3B expressions correlated with worse OS among stages I, II, III, and IV and grades 3 and 4. To explore whether TBC1D3 expression is an independent predictor of OS in KIRC, we performed univariate and multivariate Cox regression analyses. In the univariate Cox regression analysis, age, grade, stage, TM classification and TBC1D3 expression were all independent risk factors for OS (p=2.84E-04, 8.86E-16, 2.54E-21, 5.00E-15, 2.59E-17 and 3.92E-06, respectively); in the multivariate Cox regression analysis, age, grade, stage and TBC1D3 expression were independent risk factors for OS (p=0.001, 0.045, 0.002 and 0.001, respectively) (Table 2).

**Table 1:** Correlation of TBC1D3 and TBC1D3B mRNA expression and clinical prognosis in KIRC with different clinicopathological factors by Kaplan-Meier plotter

			Overall survival (530)				
		TBC1D3			TBC1D3B		
Clinicopathological characteristics		N	Hazard ratio	p value	n	Hazard ratio	p value
**Sex**							

	Female	186	2.18 (1.32–3.6)	0.0019^a^	186	1.79 (1.07–2.01)	0.024^a^
	Male	344	1.92 (1.31–2.83)	0.00075^a^	344	1.85 (1.25–2.74)	0.0018^a^

**Stage**							

	1	67	3.88 (1.1–13.74)	0.024^a^	265	1.94 (1.07–3.52)	0.025^a^
	2	57	6.57 (1.73–25.01)	0.0018^a^	57	7.72 (2.33–25.58)	9.6E-05^a^
	3	123	2.18 (1.23–3.86)	0.0061^a^	123	1.8 (1–3.26)	0.048^a^
	4	82	2.01 (1.21–3.36)	0.0064^a^	82	1.43 (0.84–2.42)	0.19

**Grade**							

	1	–	–	–	–	–	–
	2	227	1.35 (0.75–2.45)	0.32	227	2.32 (1.2–4.51)	0.01^a^
	3	206	2.37 (1.47–3.81)	0.00027^a^	206	1.72 (1.07–2.75)	0.023^a^
	4	75	1.85 (1.04–3.29)	0.033^a^	75	1.93 (1.06–3.53)	0.03^a^

a Values indicate p < 0.05

**Table 2:** Univariate and multivariate analysis of the correlation of TBC1D3 expression with OS among KIRC patients.

	Univariate analysis		Multivariate analysis	
Parameter	HR(95%CI)	P value	HR(95%CI)	P value
Age	1.771(1.304-2.405)	2.48E-04^a^	2.753(1.497-5.064)	0.001^a^
Gender	1.053(0.774-1.433)	0.741	0.778(0.397-1.524)	0.464
Grade	2.286(1.869-2.797)	8.86E-16^a^	1.608(1.011-2.558)	0.045^a^
Stage	1.883(1.652-2.146)	2.54E-21^a^	2.625(1.396-4.935)	0.002^a^
T classification	1.916(1.628-2.254)	5.00E-15^a^	0.661(0.385-1.135)	0.133
M classification	3.766(2.770-5.119)	2.59E-17^a^	0.777(0.315-1.915)	0.584
N classification	0.914(0.679-1.229)	0.552	1.138(0.302-4.284)	0.848
TBC1D3	2.071(1.521-2.822)	3.92E-06^a^	3.568(1.667-7.639)	0.001^a^

a values indicate p＜0.05

### Functions of the TBC1D3 family in KIRC

**Figure 4:**
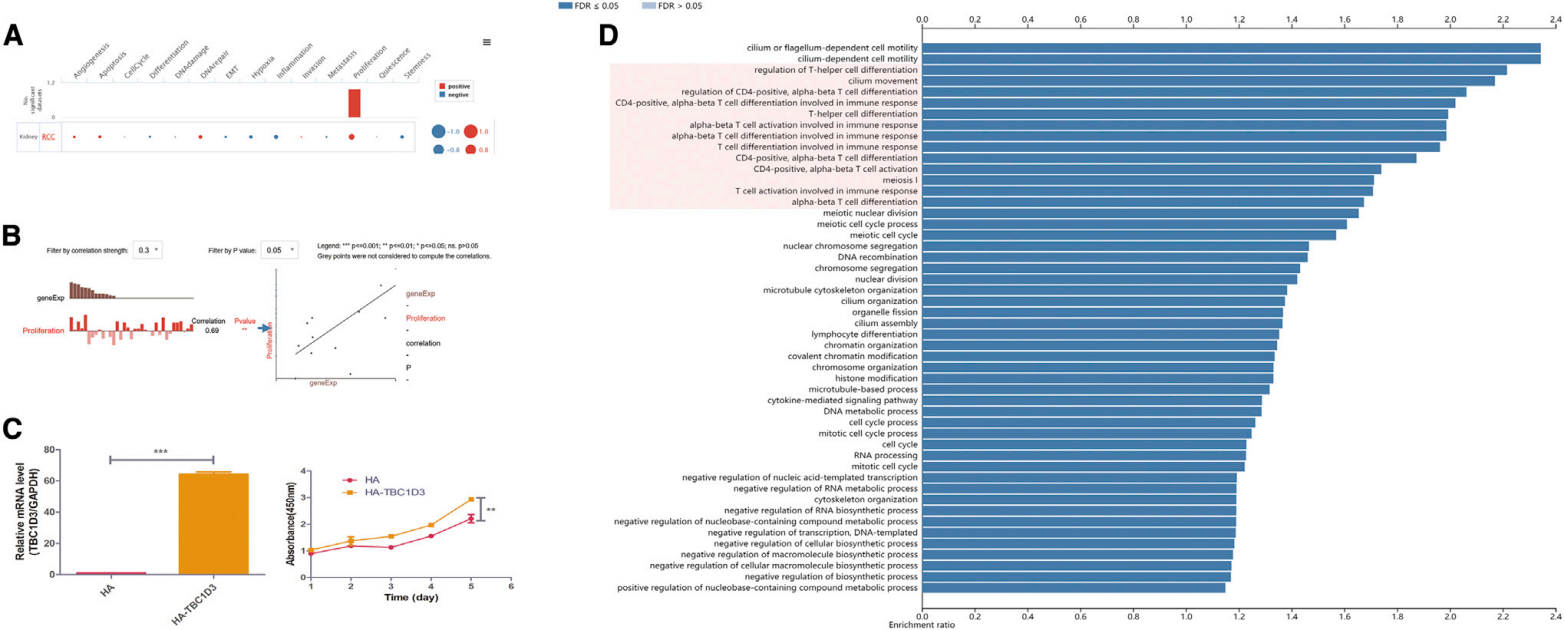
Function of the TBC1D3 family in KIRC (A) Single-cell analysis indicated that TBC1D3D was primarily involved in regulating proliferation and DNA repair. (B) Data from CancerSEA demonstrated that TBC1D3D was positively correlated with regulating proliferation. (C) Caki-1 cells were transfected with HA-TBC1D3 or control HA vector, and then we found that TBC1D3 can promote the proliferation of renal clear cell. (D) The biological process of TBC1D3 was analyzed by LinkedOmics.

To investigate the functions of the TBC1D3 family in KIRC, we performed single-cell analysis using CancerSEA. The results indicated that TBC1D3D positively regulated proliferation and negatively regulated inflammation in KIRC cells (Figures 4A and 4B). We constructed the plasmid of TBC1D3 and transfected the Caki-1 cell, the result showed that TBC1D3 can promote the proliferation of ccRCC cells (Figure 4C). The biological process of TBC1D3 was identified by overrepresentation enrichment analysis (ORA), which revealed that TBC1D3 expression was closely related to immune response (Figure 4D). According to the Human Protein Atlas (HPA) database, TBC1D3 family members are located in the membrane. In addition, TBC1D3 and TBC1D3B expressions in blood cells specifically enhance RNA levels of neutrophils and natural killer (NK) cells, respectively (Figure S2).

### The association between TBC1D3 family expression and immunoinhibitor

**Figure 5:**
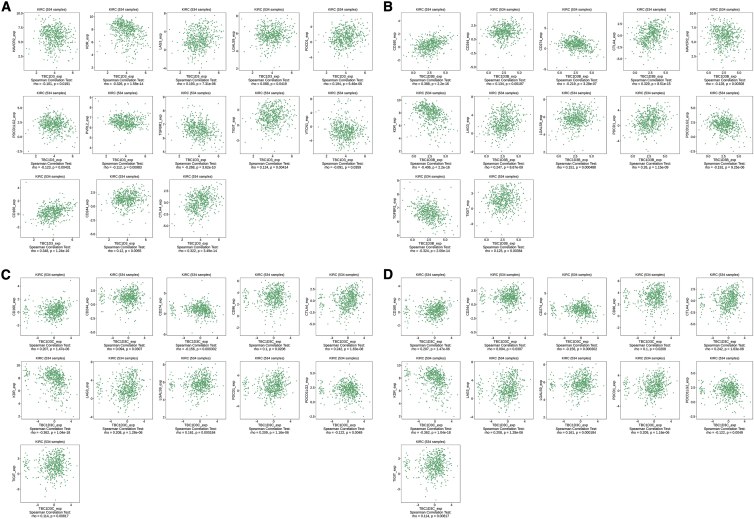
Correlations of TBC1D3 family expression and immune inhibitors in KIRC (A) TBC1D3 expression was positively correlated with CD160, CTLA4, CD244, LAG3, LGALS9, PDCD1, and TIGIT and negatively correlated with HAVCR2, PDCD1LG2, PVRL2, and TGFBR1. (B) TBC1D3B expression was positively correlated with CD160, CD244, CTLA4, LAG3, LGALS9, PDCD1, and TIGIT and negatively correlated with CD274, HAVCR2, PDCD1LG2, KDR, PVRL2, and TGFBR1. (C) TBC1D3C expression was positively correlated with CD96, CD160, CD244, CTLA4, LAG3, LGALS9, PDCD1, and TIGIT and negatively correlated with CD274, KDR, and PDCD1LG2. (D) TBC1D3H expression was positively correlated with CD160, CTLA4, CD244, LAG3, PDCD1, and TIGIT and negatively correlated with CD274, HAVCR2, IL10RB, KDR, PDCD1LG2, PVRL2, and TGFBR1.

In the past two decades, novel immune checkpoint inhibitors have made great progress with the improvement of understanding of human immune function.^8^ Consequently, we assessed whether TBC1D3 family expression was associated with immune checkpoint inhibitors. The TISIDB database was chosen to investigate the association between TBC1D3 family expression and immune inhibitory effects. As a result, TBC1D3, TBC1D3B, TBC1D3C, and TBC1D3G are four members of the TBC1D3 family that were associated with CD160, CTLA4, CD244, KDR, LAG3, PDCD1, PDCD1LG2, and TIGIT. In addition, TGFBR1 and HAVCR2 were associated with TBC1D3, TBC1D3B, and TBC1D3G. CD274 was associated with TBC1D3B, TBC1D3C, and TBC1D3G. In addition, TBC1D3 was associated with LGALS9, TBC1D3B was associated with CD274, TBC1D3C was associated with CD96 and LGALS9, and TBC1D3G was associated with IL10RB and PVRL2 (Figure 5).

### The association between TBC1D3 family expression and immune infiltration

**Figure 6:**
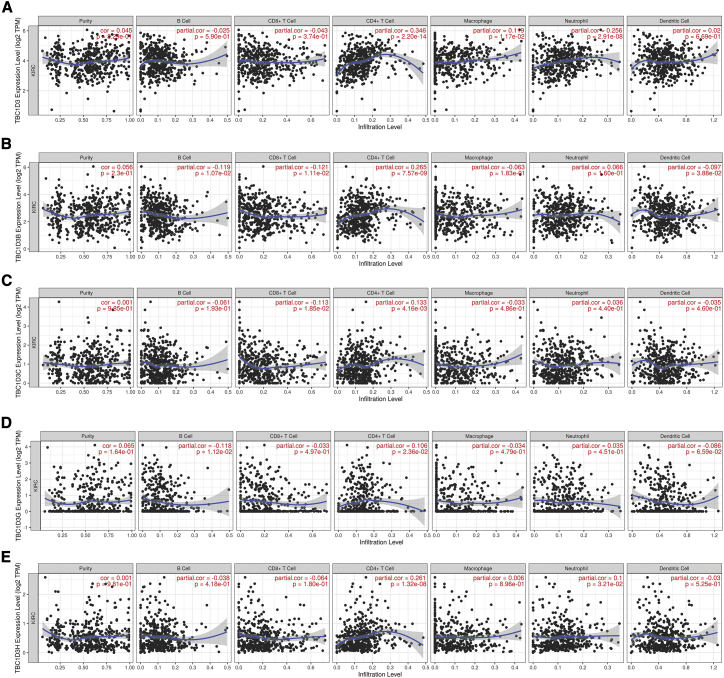
Correlation of TBC1D3 family expression with immune infiltration levels in KIRC (A) TBC1D3 expression was positively correlated with infiltration levels of CD4^+^ T cells, macrophages, and neutrophils. (B) TBC1D3B was negatively correlated with infiltration levels of B cells, CD8^+^ T cells and dendritic cells. (C) TBC1D3C was positively correlated with infiltration levels of CD8^+^ T cells and negatively correlated with CD4^+^ T cells. (D) TBC1D3G was correlated with infiltration levels of B cells, CD4^+^ T cells, and dendritic cells. (E) TBC1D3H was positively correlated with infiltration levels of CD4^+^ T cells and neutrophils.

**Figure 7:**
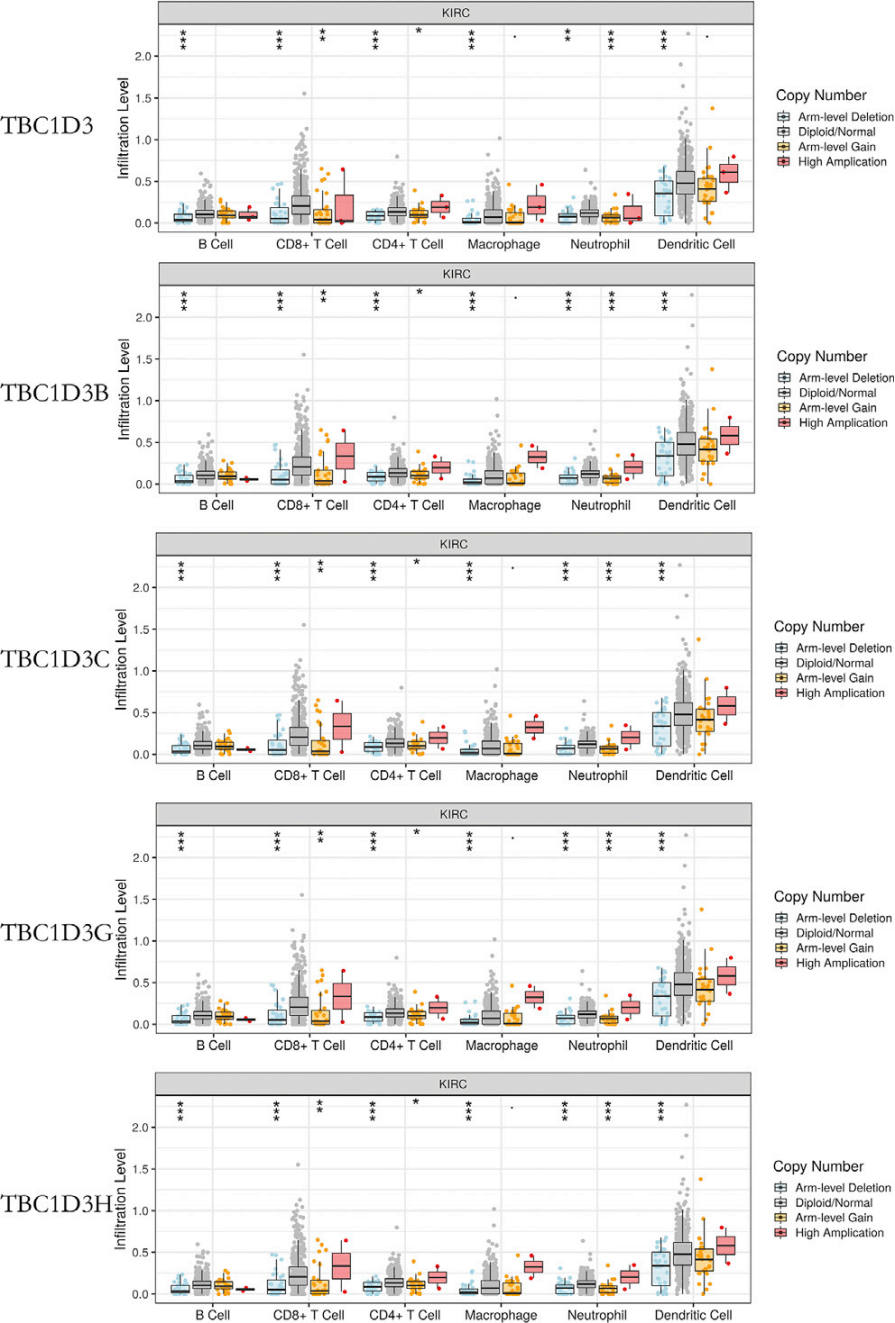
Correlation of tumor infiltrating levels in KIRC and different somatic copy numbers’ alterations in the TBC1D3 family members The arm-level deletion of TBC1D3 family members was significantly associated with decreased tumor infiltration levels in KIRC. In addition, arm-level gain of TBC1D3 family members was significantly associated with increased CD8^+^ T cells, CD4^+^ T cells, and neutrophil infiltration levels.

TIMER analysis was performed to investigate the relationship between TBC1D3 family expression and tumor-infiltrating lymphocytes in KIRC. TBC1D3 family expression was positively associated with CD4^+^ T cell infiltration levels. In addition, macrophage infiltration levels were only significantly associated with TBC1D3 expression and dendritic cell infiltration levels were negatively correlated with TBC1D3B expression. Neutrophil’s infiltration levels were found to be positively correlated with TBC1D3 and TBC1D3H expressions (Figure 6). To further confirm the relationship between TBC1D3 expression and immune cell infiltration levels in KIRC, we used TIMER to explore the correlations between TBC1D3 expression and various immune infiltration associated markers.^9^ Our results showed there was a significant correlation between TBC1D3 expression and the most of marker sets of neutrophils, Th1, Th2, Treg, and T cell exhaustion (Table 3). Especially for T cell exhaustion, the results were consistent with analysis of DISTIB. Somatic copy number alterations (SCNA) module showed that the arm-level deletion of TBC1D3 family members was significantly associated with immune cell infiltration levels in KIRC (Figure 7).

**Table 3:** Correlation analysis between TBC1D3 and relate genes and markers of immune cells in KIRC by TIMER.

	Gene markers	KIRC			
		None		Purity	
		Correlation	p	Correlation	p
CD8^+^ T cell	CD8A	0.077	ns	0.062	ns
	CD8B	0.061	ns	0.053	ns
T cell (general)	CD3D	0.072	ns	0.054	ns
	CD3E	0.101	∗	0.085	ns
	CD2	0.111	∗	0.093	∗
B cell	CD19	0.133	∗	0.117	∗
	CD79A	−0.022	ns	−0.014	ns
Monocyte	CD86	0.051	ns	0.072	ns
	CD115 (CSF1R)	0.099	ns	0.112	∗
TAM	CCL2	−0.008	ns	−0.028	ns
	CD68	0.006	ns	0.031	ns
	IL10	0.068	ns	0.18	ns
M1 macrophage	INOS (NOS2)	−0.003	ns	−0.002	ns
	IRF5	0.365	∗∗∗	0.358	∗∗∗
	COX2 (PTGS2)	−0.001	ns	−0.044	ns
M2 macrophage	CD163	0.034	ns	0.067	ns
	VSIG4	0.054	ns	0.079	ns
	MS4A4A	0.031	ns	0.049	ns
Neutrophils	CD66b (CEACAM8)	0.169	∗∗∗	0.166	∗∗∗
	CD11B (ITGAM)	0.151	∗∗∗	0.168	∗∗∗
	CCR7	0.088	ns	0.09	ns
Natural killer cell	KIR2DL1	0.022	ns	0.018	ns
	KIR2DL3	0.049	ns	0.07	ns
	KIR2DL4	0.061	ns	0.067	ns
	KIR3DL1	−0.02	ns	−0.017	ns
	KIR3DL2	0.004	ns	−0.009	ns
	KIR3DL3	−0.002	ns	−0.016	ns
	KIR2DS4	−0.027	ns	−0.029	ns
Dendritic cell	HLA-DPB1	0.011	ns	0.025	ns
	HLA-DQB1	0.056	ns	0.065	ns
	HLA-DRA	0	ns	0.02	ns
	HLA-DPA1	0.023	ns	0.032	ns
	BDCA-1 (CD1C)	0.043	ns	0.057	ns
	BDCA-4 (NRP1)	0.041	ns	0.065	ns
	CD11c (ITGAX)	0.424	∗∗∗	0.436	ns
Th1	T-bet (TBX21)	0.241	∗∗∗	0.24	∗∗∗
	STAT4	0.366	∗∗∗	0.363	∗∗∗
	STAT1	0.079	ns	0.082	ns
	IFN-γ (IFNG)	0.176	∗∗∗	0.172	∗∗∗
	TNF-α (TNF)	0.249	∗∗∗	0.245	∗∗∗
Th2	GATA3	0.062	ns	0.065	ns
	STAT6	0.357	∗∗∗	0.383	∗∗∗
	STAT5A	0.163	∗∗∗	0.177	∗∗∗
	IL13	0.36	∗∗∗	0.327	∗∗∗
T fh	BCL6	0.347	∗∗∗	0.332	∗∗∗
	IL21	0.069	ns	0.071	ns
Th17	STAT3	0.093	ns	0.012	∗∗
	IL17A	0.002	ns	−0.023	ns
Treg	FOXP3	0.2	∗∗∗	0.187	∗∗∗
	CCR8	0.187	∗∗∗	0.199	∗∗∗
	STAT5B	0.163	∗∗∗	0.177	∗∗∗
	TGF-β (TGFB1)	0.017	ns	0.011	ns
T cell exhaustion	PD-1 (PDCD1)	0.171	∗∗	0.15	∗∗
	CTLA4	0.33	∗∗∗	0.314	∗∗∗
	TIM-3 (HAVCR2)	−0.024	ns	−0.015	ns
	GZMB	0.046	ns	0.05	ns
	LAG3	0.167	∗∗∗	0.141	∗∗∗
	PDL1 (CD274)	0.106	∗∗	0.099	∗

TAM, tumor-associated macrophage; Th, T helper cell; Tfh, Follicular helper T cell; Treg, regulatory T cell; Cor, R value of Spearman’s correlation; None, correlation without adjustment. Purity, correlation adjusted by purity. ∗ p < 0.01; ∗∗ p < 0.001; ∗∗∗ p < 0.0001.

## Discussion

TBC1D3 has been reported in many cancers. KIRC has been shown to have the highest level of immune infiltration and T cell infiltration (in 19 tumor types).^2^ Previous studies showed that TBC1D3 upregulated tumor necrosis factor-α (TNF-α)-induced breast cancer cell migration.^7^ Whether TBC1D3 family expression is associated with tumor immune infiltration in KIRC remains unknown.

The current study is the first to explore the expression and prognostic values of TBC1D3 family members in KIRC. UALCAN showed that five TBC1D3 members had higher expression levels in KIRC tumor tissue compared to normal tissue. The same result can be found in the TIMER database (data not shown). Furthermore, TBC1D3 family expression was associated with all four stages of KIRC and correlated with race, gender, age, tumor grade, KIRC subtypes, and nodal metastasis status. Further investigation is needed to confirm the role of TBC1D3 family members as a putative KIRC biomarker. Analysis of data from LinkedOmics showed that high expression levels of five TBC1D3 members were correlated with poor prognosis in KIRC, which was consistent with the TISIDB analysis result. But the data analysis from the Kaplan-Meier plotter showed a contradictory result. High levels of TBC1D3, TBC1D3B, and TBC1D3E were associated with poor prognosis, while the other two TBC1D3 family members were correlated with good prognosis (Figure S3). To our surprise, the relapse-free survival analysis from the Kaplan-Meier plotter database had the opposite result compared to the OS analysis (Figure S4). Consequently, further investigation is needed to verify the conclusion. We found no proper TBC1D3 antibody after testing many antibodies on the market; in future, the expression and prognosis of TBC1D3 would be verified. However, whether the prognostic result was good or poor, TBC1D3 family expression was an effective prognostic biomarker for KIRC.

To further evaluate the function of the TBC1D3 family, we performed data analysis using GeneMANIA, STRING, and CancerSEA. CancerSEA analysis results showed that TBC1D3D may influence KIRC development and progression by regulating cell proliferation. TBC1D3 has been found to colocalize with microtubule protein,^6^ which was also shown in the HPA database. In addition, TBC1D3 was colocalized with Rab5 and Cu17, as shown in the STRING data. Previous studies have demonstrated that tubulin played a crucial role in the cell cycle and cell proliferation.^10^ Therefore, TBC1D3 may promote KIRC development and progression through binding to microtubules.

Recently, tumor-associated immune cells have attracted much attention. Generally speaking, tumor-associated immune cells consist of two types: tumor-antagonizing and tumor-promoting immune cells.^11^ Tumor-antagonizing immune cells contain effector T cells, NK cells, dendritic cells, M1-polarized macrophages, and N1-polarized neutrophils, while tumor-promoting immune cells include regulatory T cells and myeloid-derived suppressor cells (MDSCs). As for B cells, their function is controversial. CD4^+^ T cells can regulate cell proliferation in KIRC by regulating the transforming growth factor β1 (TGF-β1)/YBX1/HIF2A signal.^12^ Abundant CD8^+^ T cell correlated with prolonged prognosis in KIRC.^13^ In this study, TBC1D3 family expression was found to be positively correlated with CD4^+^ T cell infiltrating level. B cell infiltrating level expression was negatively correlated with TBC1D3B and TBC1D3G. TBC1D3 family expression may upregulate the infiltrating levels of CD4^+^ T and B cells to promote the development of KIRC.

In recent years, drug trials targeting immune checkpoints have reported significant improvements in survival rates of KIRC patients.^14^^,^^15^ TBC1D3 family expression was positively associated with CD160, CTLA4, CD244, LAG3, PDCD1, and TIGIT, while its expression was negatively correlated with KDR and PDCD1LG2. Currently, the blockade of PD-1 signaling using the PD-1/PD-L1 antibody and the blockade of CTL4 signaling using the CTL4 antibody have both shown promising therapeutic effects in a variety of cancers, such as melanoma, non-small-cell lung cancer, renal cell cancer, and lymphoma.^16^, ^17^, ^18^ Programmed cell death protein-1 (PD-1, encoded by PDCD1) is expressed in activated T cells and suppress the activation of lymphocytes and cytokine production by interacting with its ligands, PD-1 ligand-1(PD-L1, encoded by PDCD1LG1), and PD-1 ligand-2 (PD-L2, encoded by PDCD1LG2). A previous study has reported that immune inhibitors CTLA4 and LAG3 correlated with poor prognosis in KIRC; in this study, we found TBC1D3 family expression was positively correlated with CTLA4 and PD1. Therefore, TBC1D3 family expressions could possibly promote the development of KIRC by increasing PD1 expression interacting with PDCD1LG2. In addition, CD160 expression can be found in NK, NKT, CD8^+^ T cells, intraepithelial T cells, and CD4^+^ T cells in humans.^19^, ^20^, ^21^, ^22^ CD160 was found to inhibit T cells and stimulate NK cells. Reduced CD160 expression impaired NK cell function and had a poor clinical prognosis in hepatocellular carcinoma patients.^23^ Because TBC1D3 family expression was positively corrected with CD160, we speculated that TBC1D3 family expression promoted the development and progression of KIRC through increasing the function of NK cell. According to TISIDB, TBC1D3 expression was associated with immune subtype, and no apparent significant differences were observed for TBC1D3B, TBC1D3C, and TBC1D3H. However, further investigation is needed to verify the results.

In conclusion, increased TBC1D3 family expression correlated with a poor prognosis. In addition, it increased immune infiltration levels of CD4^+^ T cells, macrophages, neutrophils, and dendritic cells. Our study provides promising therapeutic targets and novel biomarkers for KIRC.

## Materials and methods

### TBC1D3 family expression level analysis

We used GSCA (http://bioinfo.life.hust.edu.cn/GSCA/#/), which is an integrated genomic and immunogenomic web-based platform for gene set cancer research,^24^ to investigate the expression of the TBC1D3 across the 33 cancer types. We used UALCAN (http://ualcan.path.uab.edu/index.html), which is an interactive web portal to perform in-depth analyses of The Cancer Genome Atlas (TCGA) gene expression data,^25^ to investigate the expression of the TBC1D3 family members across tumor and normal tissues. In addition, UALCAN was used to explore the TBC1D3 family expression in KIRC patients of different races, ages, tumor grades, and other clinicopathological features.

### TBC1D3 family genomic alterations and correlation analysis

We performed an analysis of the cBio Cancer Genomics Portal (http://cbioportal.org), which is an open-access resource for interactive exploration of multidimensional cancer genomic datasets,^26^ to analyze TBC1D3 family alterations in the TCGA KIRC sample. In addition, it was used to assess correlations among TBC1D3 family members.

### Gene-gene interaction and protein-protein interaction networks

GeneMANIA (http://genemania.org/) is a fast gene network construction and function prediction for cytoscape,^27^ and STRING (https://string-preview.org/) is used for protein-protein interaction network functional enrichment analysis, both of which were used to explore the TBC1D3 family gene and protein network.

### Survival analysis

Kaplan-Meier plotter (www.kmplot.com), an online database including gene expression data and clinical data;^28^ LinkedOmics (http://www.linkedomics.org/login.php), a publicly available portal including multiomics data from 32 TCGA cancer types;^29^ UALCAN; and TISIDB were all used to examine correlations between TBC1D3 family expression and OS.

### Single-cell analysis

We used CancerSEA (http://biocc.hrbmu.edu.cn/CancerSEA/home.jsp), which is the first dedicated database that aims to comprehensively decode functional states of cancer cells at a single-cell resolution,^30^ to explore the function of TBC1D3 family expression.

### Immunoinhibitor analysis

TISIDB (http://cis.hku.hk/TISIDB/index.php), a web portal for tumor and immune system interactions, which integrates multiple heterogeneous data types,^31^ was used to investigate correlations between TBC1D3 family members and immunoinhibitors.

### Tumor-infiltrating immune cells analysis

TIMER (https://cistrome.shinyapps.io/timer/), a web server for comprehensive analysis of tumor-infiltrating immune cells,^32^ was used to explore correlations between tumor-infiltrating immune cells and TBC1D3 family expression.

### Cell culture

Cell lines Caki-1 were cultured in McCoy’s 5A (Procell, cat#PM150710) with 10% fetal bovine serum (Procell, cat#164210-500).

### Plasmids and transfection

HA-TBC1D3/pcDNA3.0 and cell transfection have been described previously.^7^

### Cell proliferation assay

A total of approximately 2 × 10^3^ KIRC cells were plated in 96-well plates. After 1, 2, 3, 4, and 5 days of culture, cell proliferation assay was assessed by the cell counting kit-8 according to the manufacturer’s protocol.

### KIRC patient tissues

Fresh KIRC tissues from cases that were histologically confirmed and did not undergo any other treatments were obtained from the Affiliated Hospital of Jiangnan University. The study was approved by the ethics committee of the Affiliated Hospital of Jiangnan University.

### Quantitative reverse transcription polymerase chain reaction

RNA was prepared by using TRIzol (Beyotime, cat#R0016) and complementary DNA was prepared by using Prime Script RT reagent Kit (Takara Bio, cat#RR047A). GAPDH was used to normalize expression level. SYBR Select Master Mix reagent (Beyotime, cat#D7170M) was used. The primers for TBC1D3 and GAPDH are described as follows:

5′-ACAAGAGCGAGAGGACAT-3′ (sense) and 5′-AGGAGGACTGACCACATC-3′ (antisense); GAPDH: 5′- TATGACAACAGCCTCAAGAT-3′ (sense) and 5′- AGTCCTTCCACGATACCA-3′ (antisense).

### Statistical analysis

Most of analyses were conducted by using R software, the rest were analyzed by SPSS and GraphPad Prism 6.0. Univariate and multivariate analysis were used to assess the influence of clinical variables on survival. Two-tailed p values less than 0.05 were considered statistically significant.

## Supplemental information

Document S1. Table S1 and Figures S1–S4

Document S2. Article plus supplemental information
