# Supplementary material for: TBC1D3 family is a prognostic biomarker and correlates with immune infiltration in kidney renal clear cell carcinoma
Source: Mol Ther Oncolytics. 2021 Jul 10;22:528–38. doi: 10.1016/j.omto.2021.06.014 (PMC8433061; doi:10.1016/j.omto.2021.06.014)

**OMTO, Volume *22***

## Supplemental information

# TBC1D3 family is a prognostic biomarker and correlates with immune infiltration in kidney renal clear cell carcinoma

**Bei Wang, Dandan Chen, and Haiying Hua**

Supplementary table 1. TBC1D3 family expression based on Race, Gender, Age, Tumor grade, ccRCC subtypes and Nodal metastasis status

| | | | Sig | | |
|---|---|---|---|---|---|
| | TBC1D3 | TBC1D3B | TBC1D3C | TBC1D3G | TBC1D3H |
| Race | | | | | |
| Normal vs Caucasian | **1.00E-12** | **1.62E-12** | **1.62E-12** | **1.62E-12** | **1.62E-12** |
| Normal vs AfricanAmerican | **8.48E-08** | **5.49E-10** | **2.13E-05** | **1.38E-04** | **1.38E-04** |
| Normal vs Asain | **1.28E-02** | 1.03E-01 | 5.08E-02 | 2.57E-01 | 2.57E-01 |
| Caucasian vs AfricanAmerican | 8.31E-01 | **3.02E-02** | **4.79E-02** | **1.89E-02** | **1.89E-02** |
| Caucasian vs Asian | **1.03E-02** | 2.57E-01 | 4.38E-01 | 3.85E-01 | 3.84E-01 |
| AfricanAmerican vs Asian | **3.81E-02** | 3.80E-01 | 7.40E-01 | 6.53E-01 | 6.53E-01 |
| Gender | | | | | |
| Normal vs Male | **1.62E-12** | **1.00E-12** | **1.00E-12** | **3.09E-12** | **3.09E-12** |
| Normal vs Female | **1.62E-12** | **1.00E-12** | **3.13E-11** | **6.97E-09** | **6.97E-09** |
| Male vs Female | 1.34E-01 | 3.24E-01 | 8.70E-01 | 1.54E-01 | 1.54E-01 |
| Age | | | | | |
| Normal vs Age(21-40Y) | **3.02E-04** | **7.97E-05** | **5.96E-04** | 1.18E-01 | 1.18E-01 |
| Normal vs Age(41-60Y) | **1.00E-12** | **1.62E-12** | **1.00E-12** | **6.99E-09** | **6.99E-09** |
| Normal vs Age(61-80Y) | **1.00E-12** | **1.62E-12** | **8.88E-16** | **2.63E-11** | **2.62E-11** |
| Normal vs Age(81-100Y) | **6.01E-05** | **4.78E-04** | 6.63E-02 | 6.04E-02 | 6.04E-02 |
| Age(21-40Y) vs Age(41-60Y) | 7.71E-01 | 9.60E-01 | 3.51E-01 | 1.07E-01 | 1.07E-01 |
| Age(21-40Y) vs Age(61-80Y) | 9.92E-01 | 8.30E-01 | 2.30E-01 | 5.29E-02 | 5.29E-02 |
| Age(21-40Y) vs Age(81-100Y) | 7.14E-01 | 6.00E-01 | 7.97E-01 | 3.96E-01 | 3.96E-01 |
| Age(41-60Y) vs Age(61-80Y) | 5.65E-01 | 6.50E-01 | 3.11E-01 | 6.42E-01 | 6.42E-01 |
| Age(41-60Y) vs Age(81-100Y) | 8.29E-01 | 5.20E-01 | 5.44E-01 | 9.01E-01 | 9.01E-01 |
| Age(61-80Y) vs Age(81-100Y) | 6.88E-01 | 4.10E-01 | 4.40E-01 | 7.43E-01 | 7.43E-01 |
| Tumor grade | | | | | |
| Normal vs Grade 1 | **5.88E-03** | **9.44E-04** | **2.44E-03** | 1.33E-01 | 1.33E-01 |
| Normal vs Grade 2 | **1.62E-12** | **1.00E-12** | **1.62E-12** | **1.47E-07** | **1.46E-07** |
| Normal vs Grade 3 | **1.00E-12** | **1.00E-12** | **1.62E-12** | **4.42E-09** | **4.42E-09** |
| Normal vs Grade 4 | **2.47E-10** | **9.09E-11** | **2.80E-06** | **2.03E-05** | **2.03E-05** |
| Grade 1 vs Grade 2 | 3.15E-01 | 3.80E-01 | 9.23E-02 | 5.77E-01 | 5.77E-01 |
| Grade 1 vs Grade 3 | 4.63E-01 | 8.50E-01 | 2.41E-01 | 9.04E-01 | 9.04E-01 |
| Grade 1 vs Grade 4 | 6.88E-01 | 6.70E-01 | 4.44E-01 | 7.99E-01 | 7.99E-01 |
| Grade 2 vs Grade 3 | 4.55E-01 | 1.00E-01 | 4.55E-01 | 2.47E-01 | 2.47E-01 |
| Grade 2 vs Grade 4 | 2.92E-01 | 2.40E-02 | 3.64E-01 | 8.05E-02 | 8.05E-02 |
| Grade 3 vs Grade 4 | 6.06E-01 | 1.77E-01 | 6.36E-01 | 4.16E-01 | 4.16E-01 |
| ccRCC subtypes | | | | | |
| Normal vs ccA subtype | **1.11E-16** | **1.62E-12** | **4.44E-16** | **3.75E-07** | **3.75E-07** |
| Normal vs ccB subtype | **1.11E-16** | **1.62E-12** | **1.62E-12** | **1.95E-08** | **1.95E-08** |
| ccA subtype vs ccA subtype | 2.91E-01 | **1.46E-03** | **4.46E-02** | **2.62E-03** | **2.62E-03** |
| Nodal Metastasis status | | | | | |
| Normal vs N0 | **1.00E-12** | **1.62E-12** | **5.59E-14** | **5.45E-10** | **5.45E-10** |
| Normal vs N1 | **1.52E-03** | **1.03E-03** | **1.47E-02** | **2.56E-02** | **2.56E-02** |
| N0 vs N1 | 2.58E-01 | 3.21E-01 | 3.08E-01 | 7.52E-01 | 7.52E-01 |

Supplementary Figure 1. Expression of TBC1D3 Mrna in KIRC tissues. T: KIRC tissues; N: noncancerous tissues.

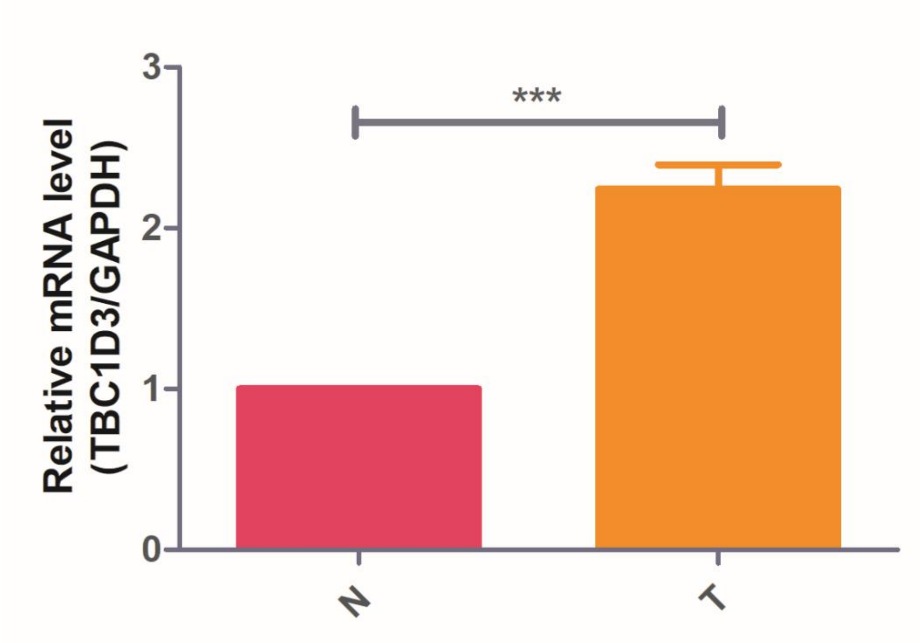


Supplementary Figure 2. The Human Protein Atlas database revealed that TBC1D3 was colocalized with microtubule proteins in the cytoplasm of U-2 OS and U-251 MG cells.

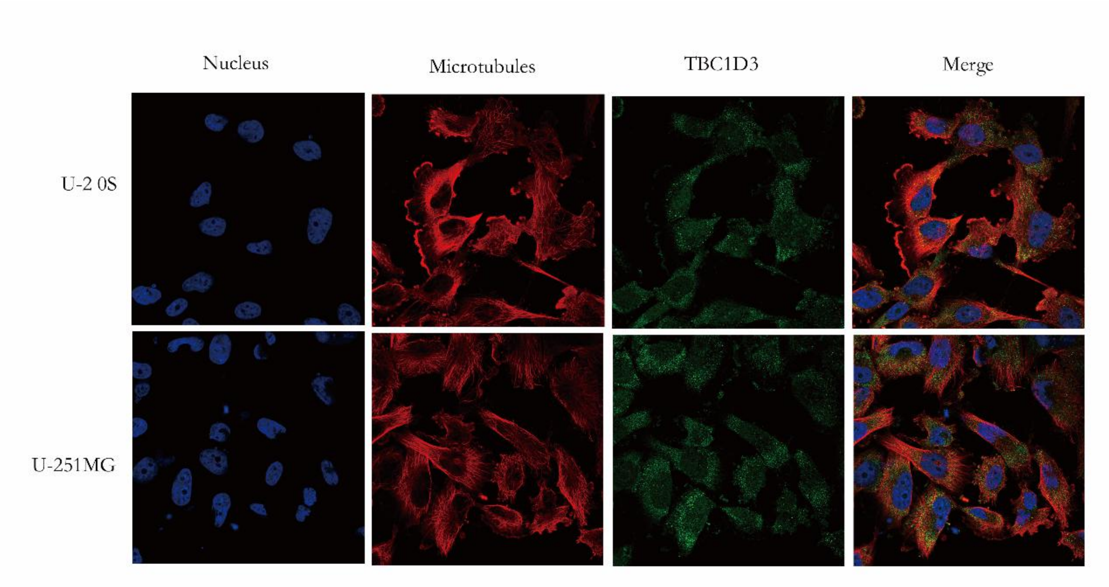


Supplementary Figure 3. Overall survival comparing the high and low expression of TBC1D3 family members in KIRC.

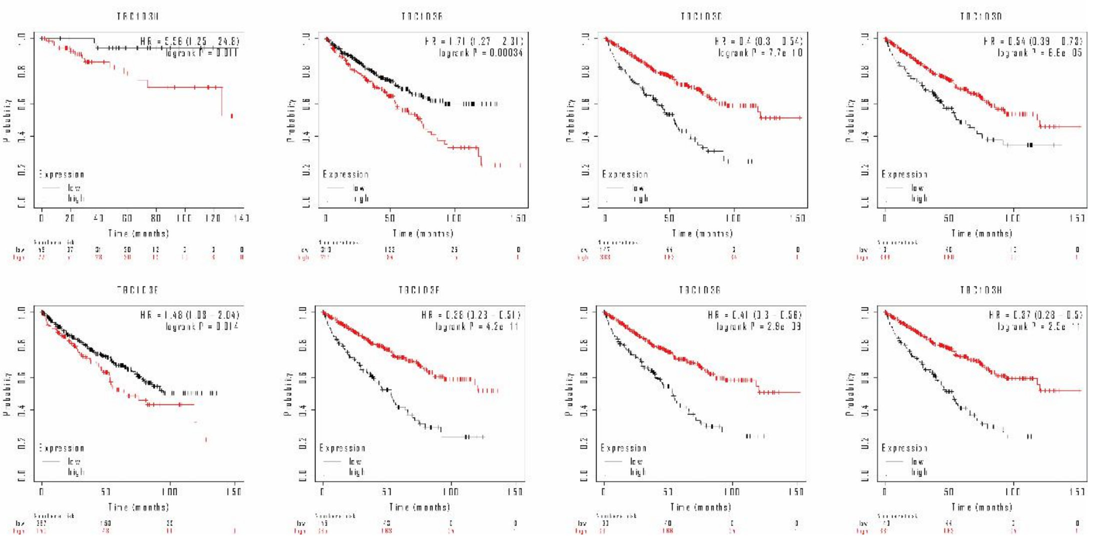

Supplementary Figure 4. Relapse-free survival comparing the high and low expression of TBC1D3 family members in KIRC. High levels of TBC1D3, TBC1D3B were associated with better prognoses while the other TBC1D3 family members were correlated with poor prognoses.

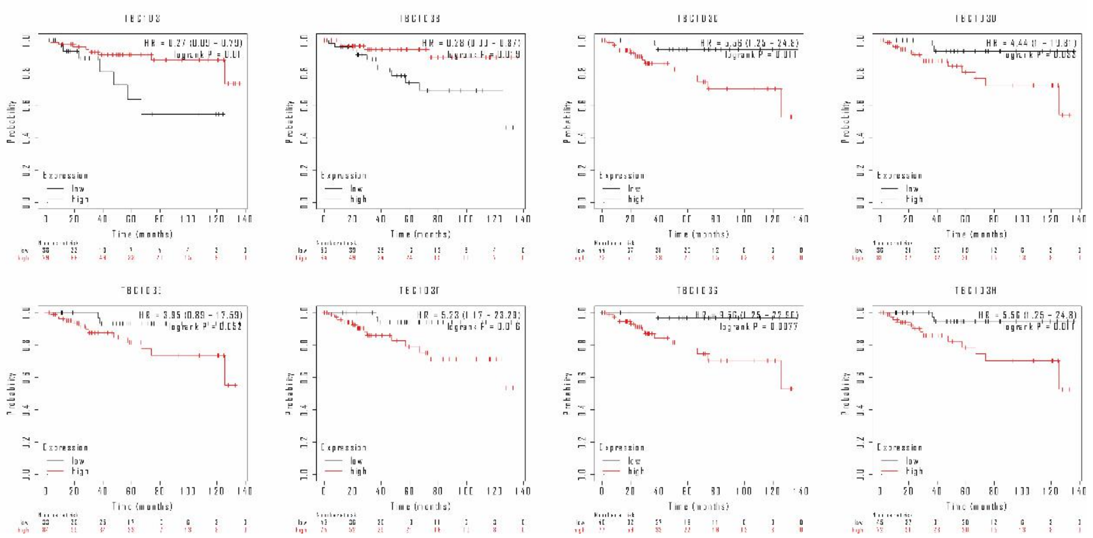

Supplement: Document S1. Table S1 and Figures S1–S4 [file mmc1.pdf]
